# Plasticity of the Human Olfactory System: The Olfactory Bulb

**DOI:** 10.3390/molecules180911586

**Published:** 2013-09-17

**Authors:** Caroline Huart, Philippe Rombaux, Thomas Hummel

**Affiliations:** 1 Department of Otorhinolaryngology, Cliniques Universitaires Saint-Luc, Brussels 1200, Belgium; 2 Institute of Neuroscience, Université catholique de Louvain, Brussels 1200, Belgium; E-Mail: philippe.rombaux@uclouvain.be; 3 Department of Otorhinolaryngology, Technical University Dresden Medical School, Dresden 01307, Germany; E-Mail: thummel@mail.zih.tu-dresden.de

**Keywords:** olfaction, olfactory bulb, plasticity

## Abstract

In the last years, an increasing interest has been paid to the olfactory system, particularly to its abilities of plasticity and its potential continuous neurogenesis throughout adult life. Although mechanisms underlying adult neurogenesis have been largely investigated in animals, to some degree they remain unclear in humans. Based on human research findings, the present review will focus on the olfactory bulb as an evidence of the astonishing plasticity of the human olfactory system.

## 1. Introduction

Olfaction plays a major role in our interaction with the environment. The olfactory system not only acts for the detection of potential dangers in the environment, such as smoke, gas or dusts, but also it influences our nutrition, social behavior, and well-being. The olfactory bulb (OB) plays the central role in the processing of olfactory information. It is the only relay between periphery and the central nervous system; it also processes olfactory information. 

The OB volume varies as a function of olfactory sensitivity and is decreased in patients with olfactory disorders (*i.e.*, post-infectious, post-traumatic, or sinunasal olfactory loss) [1,2,3,4,5]. But even more interestingly, the OB volume may increase during recovery from the olfactory disorder, highlighting its plasticity [6].

It has been hypothesized that this plasticity could be due to the particularity of the olfactory system that is continuous neurogenesis throughout adult life (e.g., [7]). Two major mechanisms of neurogenesis have been proposed–and they are still under discussion (e.g., [8,9]). The first one is the continuous renewal of olfactory receptor neurons (ORNs) from basal cells at the level of the olfactory neuroepithelium and the synaptogenesis that occurs between the axons of ORNs and mitral cells at the glomerular level. The second one is the continuous neurogenesis from the subventricular zone of the lateral ventricle, leading to the generation of neuroblasts that migrate along the rostral migratory stream and that will differentiate into interneurons inside the OB.

For years adult neurogenesis has been a topic of high interest. If adult neurogenesis has been largely investigated in animals, only few studies have investigated the neurogenesis in humans. However, the animals and human olfactory system show noticeable differences and extrapolation of animal studies to humans might be too simplistic and misrepresent the reality. Focusing on human findings, the present review attempts to discuss the plasticity within the human OB. 

## 2. Anatomy and Physiology of the OB

**Figure 1:**
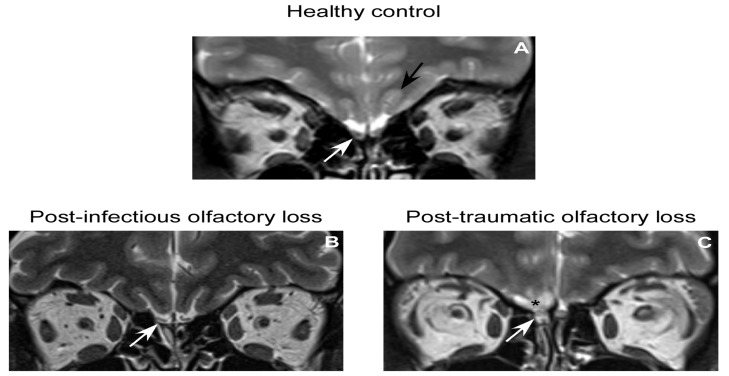
Coronal T2-weighted images of the olfactory bulbs (OBs) in normal subject (**A**) and in patients suffering from post-infectious (**B**) and post-traumatic (**C**) olfactory loss.

The OB is ovoid in shape and located in the anterior cranial fossa, above the cribriform plate of the ethmoid bone, under the frontal lobe (Figure 1). It receives axons from the olfactory receptor neurons (ORNs), which pass through the cribriform plate of the ethmoid bone; converge into the olfactory nerves, surrounded by glial cells (called olfactory ensheating cells) and project directly to the ipsilateral OB. 

The OBs (white arrow) are located above the cribriform plate of the ethmoid bone, under the frontal lobes. The olfactory sulcus is indicated by a black arrow. The OB is decreased in patients suffering from post-infectious olfactory loss (**B**) as well as in patients suffering from post-traumatic olfactory loss (**C**). Moreover, in the later OB is fragmented and basifrontal contusions may be observed (asterisk).

ORNs are bipolar cells, with their body located in the olfactory neuroepithelium and their dendritic extensions directed toward the olfactory cleft, carrying on their surface several cilia surrounded by olfactory mucus. Olfactory neuroepithelium is special in the sense that it is continuously regenerated throughout adulthood due to basal cell. 

Odorants reaching the olfactory cleft are probably carried through the mucus layer by olfactory binding proteins; and bind to olfactory receptors located at the ORNs’ cilia. In 1991, Axel and Buck [10] discovered a family of approximately 1,000 genes that encode for an equivalent number of olfactory receptors, corresponding to the largest family of genes in the mammalian genome [11], highlighting their important role in physiology. In the majority of mammals most of these genes are functional, but in primates the number of functional genes decreases and is only about 350 in humans [12]. Axel and Buck found that each ORN possesses only one type of odorant receptor and each receptor is specialized for a small number of odors. Hence, a given odorant will bind a typical pattern of olfactory receptors. 

In the OB, ORNs axons ramify and synapse with second order neurons (named mitral cells) into spherical structures known as glomeruli. Each glomerulus collects axons of ORNs that express the same receptor protein [13]. Glomeruli are major structures within the OB and can be considered to be the first olfactory structure, relaying directly the peripheral olfactory information to the central nervous system. 

The OB has a laminar organization arranged in circular layers. It encompasses six different layers, anatomically defined on the basis of cell type and composition: (1) the external or olfactory nerve layer is made up of axons of the incoming ORNs; (2) the glomerular layer is composed by glomeruli wherein axons of ORNs synapse with dendrites of mitral cells, periglomerular and tufted cells; (3) the external plexiform layer consists mainly of dendrites of mitral and tufted cells. Indeed, mitral and tufted cell extend secondary dendrites into this layer, where they synapse with local interneurons (juxtaglomerular, periglomerular and granule cells) (4) the mitral cells layer contains cell bodies of mitral cells (second order olfactory neurons); (5) the internal plexiform layer; and (6) the granule cell layer contains soma of the granule cells, which are GABAergic cells and represent the most numerous cells in the OB.

Axons of the mitral cells and tufted cells coalesce to form the olfactory tract, located at the base of the forebrain. The olfactory tract conveys olfactory information to a wide number of brain regions within the frontal lobe and the dorsomedial surface of the temporal lobe, often referred to as primary olfactory cortex. 

**Figure 2:**
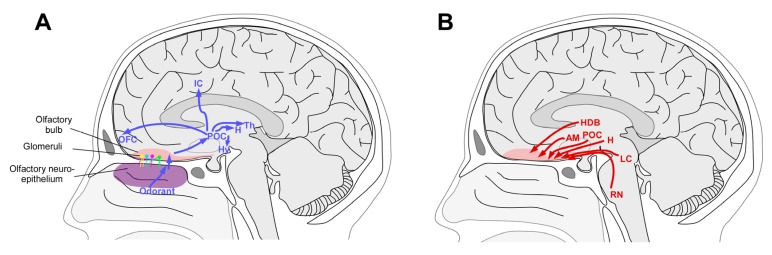
Centripetal (**A**) and centrifugal (**B**) information from and to the olfactory bulb.

This centripetal information then projects to the primary olfactory cortex (Figure 2). Glutamate is the principal neurotransmitter of the ORNs, mitral and tufted cells. Dopamine and GABA receptors are present on the receptor cells, allowing presynaptic modulation of the glutamate output by the interneurons [14,15]. However, it is important to note that numerous neurotransmitters are involved in bulbar cell interactions at the level of the glomerulus and within the external plexiform layer (for a review, see [16]).

**Figure 3:**
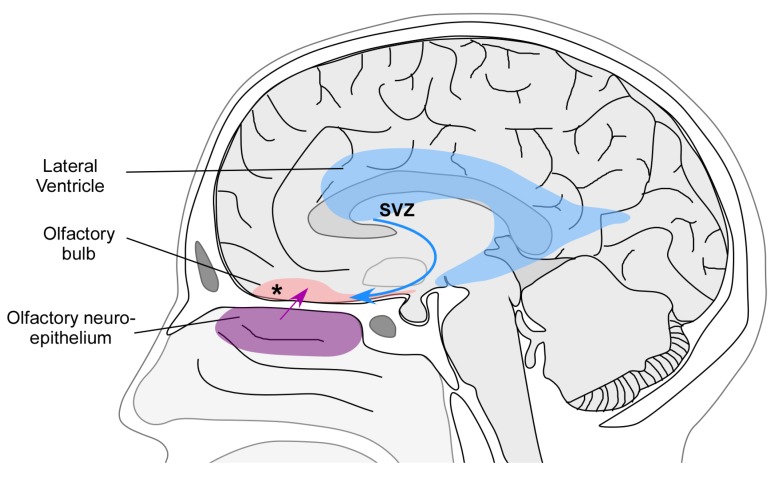
Mechanisms underlying the neurogenesis and plasticity of the olfactory bulb.

OB also receives centrifugal information, from higher structures of the brain (Figure 3). Centrifugal fibers, with GABA and acetylcholine as principal neurotransmitters, are essential to modulate the activity of the OB. Cholinergic fibers enter the bulb from the ispilateral nucleus of the horizontal limb of the diagonal band [17,18]. Centrifugal serotoninergic innervation from the dorsal and medial raphe nuclei, noradrenergic innervation from the locus coeruleus and glutamatergic innervation form the anterior olfactory nucleus are also present (for a review, see [16]). Interestingly, it has been demonstrated that the centrifugal projections from noradrenergic neurons located in the locus coeruleus is critical in early olfactory preferences learning, both in rodents [19,20] and humans [21,22]. In animals, it has been demonstrated that centrifugal fibers contribute to the context-dependent modulation of the OB activity and affect olfactory learning, memory, attention and odor-reward association (for a review see [23]).

(**A**): Odorants reaching the olfactory cleft stimulate the olfactory receptor neurons (ORNs) located in the olfactory neuroepithelium. Axons of ORNs synapse with second order neurons at the level of the olfactory bulb (OB), and ORNs carrying the same odorant receptor project to the same glomeruli. The olfactory information is then transmitted to the primary olfactory cortex (POC) (composed by piriform cortex, entorhinal cortex, periamygdaloid cortex, anterior olfactory nucleus, olfactory tubercle). Primary olfactory cortex then projects, among other areas, to the orbitofrontal cortex (OFC), the insular cortex (IC), thalamus (Th), Hippocampus (H) and Hypothalamus (Hy); (**B**): The OB receives centrifugal information, for example, from the horizontal limb of the diagonal band (HDB), the amygdala (AM), the primary olfactory cortex (POC), the hippocampus (H), the locus coeruleus (LC) and the raphe nuclei (RN).

Two major mechanisms have been proposed to explain the plasticity of the olfactory system. **First**, the continuous neurogenesis that occurs at the level of the olfactory neuroepithelium (purple). **Second**, the continuous neurogenesis from the subventricular zone of the lateral ventricle (SVZ). An additional **possible mechanism** relates to the intrinsic bulbar plasticity (asterisk).

In summary, the neuronal activity within the OB depends on sensory input from the olfactory epithelium (centripetal input) and centrifugal input from the olfactory cortex (for a review see [23]). The OB is not only a relay conveying olfactory information to the central nervous system, but it appears to process actively the olfactory information and to perform complex neuronal computations, similar to those of the primary cortices of other sensory systems [24].

## 3. Mechanisms of Plasticity

Mechanisms of neurogenesis and plasticity have been extensively investigated in animals, particularly in rodents. However, there is still a controversy as to whether the mechanisms described in animals are relevant in humans. Two major mechanisms have been proposed to explain the plasticity of the OB. The first one is the continuous neurogenesis that occurs at the level of the olfactory neuroepithelium. It has been demonstrated that the olfactory epithelium contains a population of proliferating progenitor cells, located in the basal layer of the olfactory neuroepithelium and the lamina propria. These stem cells have the ability to produce neurons as well as their ensheatment and supporting cells. Hence, the olfactory neuroepithelium is continuously reconstituted and the olfactory nerves regenerate throughout life (for a review see [25]). This regeneration is of primary importance since the olfactory receptor neurons are in direct contact with the environment and hence are exposed to several potential sources of damage including toxins, infections, or trauma (Figure 3).

A second mechanism possibly explaining the plasticity is the continuous neurogenesis from the supraventricular zone (SVZ) of the lateral ventricle (Figure 3). In adult rodents and in monkeys, neural stem cells residing in the walls of the lateral ventricle give rise to neuroblasts [26,27,28]. Neuroblasts form a migratory chain, following the rostral migratory stream (RMS) and migrate toward the OB where they differentiate into olfactory interneurons throughout adult life [29,30]. These new neurons are thought to be implicated in complex processes, such as olfactory memory formation, odor discrimination and social interactions [23,31]. 

In adult human brain, neural stem cells lining the lateral ventricle have been described [32,33], but their potential role and the question as to whether they give rise to neuroblasts that migrate to the OB is still a matter of debate [33,34].

A ventral extension of the lateral ventricle suggests the presence of a RMS, where the SVZ remains an active proliferative region. Nevertheless, the existence of a human RMS and the presence of migratory neuroblasts in adults are debated. It has been proposed that neuroblasts migrate through the OB via the olfactory ventricle [34]. Although this structure seems to exist in the fetal human brain [35], recent research [36,37] does not support the idea of a persistent ventricular lumen connecting the lateral ventricle to the OB in adult humans as well as in postnatal infants. 

In fetal human brain, a RMS has been described, from the lateral SVZ to the olfactory tract and OB; as well as the presence of migrating neuroblasts [36,37]. Moreover, some neuroblasts formed chains within the RMS, similarly to chains of migrating neuroblasts identified in the SVZ and RMS of rodents and monkeys [37]. In infant humans, many proliferating cells are observed in the SVZ. However, recent studies have shown that the postnatal neurogenesis in the human adult OB may be limited [8,36,37,38], with numbers of proliferating cells and migrating neuroblasts decreasing from birth to month 18 [36].

In adult humans, there is no evidence of neuroblasts forming chains in the SVZ or the RMS. However, studies have described the presence of very few migrating neuroblasts in the SVZ and the RMS-like pathway [36,37]. Nevertheless, based on the fact that neuroblasts actively control the formation and maintenance of their own route [39], Wang *et al.* suggested that it is difficult to imagine that a small number of neuroblasts could establish a long and complex migratory route from the SVZ to the OB [37]. Moreover, in contrast to what has been described in rodents and monkeys, there seem to be little or no neuroblasts in the adult human olfactory tract or OB [37].

Hence, the SVZ maintains the ability to produce neuroblasts in the adult human brain; and a smaller and morphologically different RMS-like pathway seems to exist in adult humans [33,34,36,37]. But clear evidence of migration of neuroblasts from the SVZ to the OB is lacking and some authors suggest that, due to the very few number of neuroblasts present in the RMS-like pathway, establishing a migratory route from the SVZ to OB is almost impossible [37]. 

Recently, Lötsch *et al.* analyzed the transcriptome of adult human olfactory bulbs. Interestingly, they reported that a fifth of genes expressed in adult human olfactory bulbs serve functions of nervous system or neuron development. Although this study doesn’t answer the question of the origin of the human neurogenesis, it supports the existence of neurogenesis in the adult human olfactory bulb [7].

Finally, another possible mechanism of plasticity is intrinsic bulbar plasticity, due to the presence of progenitor cells directly within the OB itself. Such neural stem cells have been isolated from the OB of adult rodents [40] and adult patients [41] (Figure 3). 

Taken together, these studies suggest that adult human OB is a plastic structure. Few neuroblasts seem to be present in SVZ of adult humans. However, whether these neuroblasts are able to reach the OB is controversial. In addition to the two main mechanisms described, it seems reasonable to think that other actors are involved in the plasticity of the OB. Similarly to animal studies, intrinsic bulbar plasticity [40,41], or centrifugal projections from noradrenergic neurons located in the locus coeruleus are possible candidates [42,43]. Nevertheless, several studies have shown that the human olfactory system exhibits notable differences as compared to animals. Hence, it is difficult to extrapolate results from animals to humans and further research is necessary to elucidate the mechanisms of the plasticity of the OB. At present, the debate about a possible ongoing OB neurogenesis in humans is still open.

## 4. Plasticity of the Human OB

On a macroscopic level, due to the increased use of the MRI in research and diagnosis in clinics, it has become possible to assess easily the OB in humans. Although results of studies investigating the cellular mechanism of OB plasticicty in humans are controversial, MRI studies agree that the human OB is a highly plastic structure whose volume relates to olfactory function. We will describe the results of these different studies, as evidence of OB plasticity in humans.

### 4.1. OB Volume as Measure of OB Function in Humans–Technical Details

MRI is the imaging modality of choice in order to measure OB volume. Standard protocol usually includes 2-mm-thick T2-weighted images in Fast Spin Echo (FSE) mode in the coronal plane, which is the best suitable technique for anatomical olfactory tract overview, detection of parenchymal lesions and OB volumetry. OB measurement is usually performed using a 1.5T MRI, or better 3T. When evaluating patients suffering from olfactory disorders, whole brain coverage remains mandatory for detecting parenchymal lesions/processes. Hence, fluid-attenuated inversion recovery (FLAIR) sequence and hemosiderin-sensitive gradient echo T2* sequences covering the whole brain are usually performed to detect post-contusion gliotic changes (on FLAIR images) and post-traumatic hemosiderin deposits (on GRE-T2* images).

Volumetric measurement of the OB is usually performed using planimetric manual contouring. All frontal 2-mm-thick slices (without interslice gap) of the FSE T2-weighted sequence are browsed from anterior to posterior. The first image in which the OB becomes clearly recognizable is considered to be the first slice through the OB. The OB surface, calculated in mm^2^, is delineated using an electronic cursor. The surfaces on all slices are summed and the total surface is multiplied by the thickness of the slices (usually 2-mm) to give a volume in mm^3^. While the anterior part of the OB is easy to assess, in contrast the posterior end of the OB is sometimes difficult to measure. It is usually defined as a sudden decrease in the diameter of the OB, meaning that the OB ends with the olfactory tract [44]. Nevertheless, there is no clear definition of this posterior end, which might explain some differences in the measurement of OB volumes obtained by different authors [45,46,47]. Buschhüter *et al.* [46] have proposed normative data of OB volume based on data of 125 patients. They proposed that people <45 years should have a minimum OB volume of 58 mm^3^; and people >45 years should have a minimum OB volume of 46 mm^3^. 

### 4.2. OB in Healthy Subjects

The OB volume is intimately correlated to olfactory function, independently of age. Using the Sniffin’ Sticks test, Buschhüter *et al.* demonstrated that OB volume correlated significantly with overall olfactory function, measured by the TDI score; specifically, OB volume was found to correlate to specific olfactory functions namely odor thresholds and odor identification [46]. In healthy subjects it has been demonstrated that OB volume varies as a function of (1) sex, with men having a larger OB volume as compared to women and (2) age, with OB volumes decreasing significantly with advancing age. As with other senses the olfactory function decreases over time and it has been described in numerous previous studies that there is a strong decrease in olfactory function above the age of 55 years [48,49]. Using MRI, Buschhüter *et al.* [46] showed that the OB volume declines in parallel to smell function.

Several mechanisms have been proposed to explain this age-related olfactory dysfunction. At a peripheral level, changes in mucociliary movement, mucus composition, submucosal blood flow, epithelia thickness might disturb the transport of the odorant to the receptor [50]. At the level of the neuroepithelium it is assumed that the regeneration of olfactory receptor neurons decreases with age [51,52]. Moreover, studies have described a decreased extent of the olfactory epithelium [53] and a decreased density and complexity of adrenergic innervation within the lamina propria of the olfactory neuroepithelium [54]. At the level of the OB, post mortem studies have shown that the number of mitral cells continuously decreases with age, as well as the number of glomeruli, the glomerular layer thickness and the mitral cell size and concentration [55]. The number of mitral cells and glomeruli declines steadily with age at an approximate rate of 10% per decade [56]. Moreover, 86% of normal aged subjects have neurofibrillary tangles in the OB, and one third of them show amyloid deposition in the OB [57]. It has also been described that aging is associated with structural abnormalities of the OB, with olfactory nerve fibers entering deeper parts of the OB and form glomeruli outside the glomerular layer. These misrouted olfactory fivers and ectopic glomeruli might alter the normal synaptic organization and hence olfactory processing [57,58].

At a more central level, brain damage due to chronic ischemia or systemic disorders might also be proposed as a potential cause of age-related olfactory disorder. It was also shown that normal aging is associated with the presence of neurofibrillary tangles and senile plaques in the brain [30] and abundant tau pathology is present in almost one third of non-demented older people [59].

Interestingly, Hummel *et al.* reported that there is a differential change of olfactory functions with aging. Indeed, olfactory thresholds decrease more strongly with age as compared to odor discrimination and odor identification [48,60]. Since threshold measurements best reflect the function of the peripheral olfactory system than other olfactory tests [61,62,63], this finding may indicate that age-related change of olfactory function is, at least in part, due to damage of the olfactory epithelium [48]. Nevertheless, it is important to keep in mind that age-related decrease of olfactory function might also be a consequence of side effects of drugs, onset of neurodegenerative diseases…

Using MRI, Smitka *et al.* [64] showed that 59% of healthy human subjects had a central lucency in the OB, interpreted as an olfactory bulb ventricle (OBV). On contrast, autopsy results identified an OBV in only 7% of cadavers. They explained this discrepancy between MRI and histopathology by postmortem resorption of cerebrospinal fluid from OBVs. However, a later study did not verify this finding, since they found such a structure in only 5.5% [65]. More recently, an *in vitro* study on human cadavers investigated OB lamination pattern using a high resolution MRI at 3T and MR microscopy at 9.4T. This study indicated that 58.9% of images in T2 had a central hyperintensity. Nevertheless, this was not an OBV but this was due to the lamination pattern of the OB [66]. 

### 4.3. OB in Patients

The study of patients suffering from olfactory dysfunction has offered some insights into the plasticity of the human OB. It has been demonstrated that the decreased olfactory function is associated with decreased OB in patients suffering from a wide range of pathologies (post-traumatic olfactory disorder [2,4,5], post-infectious olfactory disorder [1,3] (Figure 1), sino-nasal related olfactory disorders [67], idiopathic olfactory loss [68], neurodegenerative diseases [69,70,71], acute depression [72], post total laryngectomy patients [73]). More interestingly, the recovery of olfactory function is associated with an increase in OB volume suggesting that OB volume is a highly plastic structure [6].

Most of these studies suggest that the plasticity of the OB relates to centripetal influences, meaning that the OB volumes decreases secondary to missing input, either following postinfectious olfactory loss [1,3], head trauma [2,4,5], sinonasal inflammation [67] or total laryngectomy [73]. The authors base their assumption of centripetal influences on the following findings: (1) In patients suffering from post-infectious [1,3], post-traumatic [2,4,5] and sino-nasal related olfactory loss [67], it has been demonstrated that OB volumes were reduced as compared to the normosmic population; (2) Follow up of these patients showed that changes in odor threshold correlated significantly with changes in OB volume [6,74]. Since odor threshold is more closely related to peripheral olfactory function [61,63] in comparison to odor identification or odor discrimination, this suggests that OB function is related to peripheral input rather than central input; (3) It has recently been shown that the migration of nasally administrated Thallium-201 was reduced in patients suffering from post infectious olfactory loss, post traumatic olfactory loss and chronic rhinosinusitis, as compared to healthy controls, suggesting a decreased connectivity in patients. Moreover, the migration of Thallium-201 to the OB was correlated with odor threshold as well as with OB volume [75]; (4) Finally, a recent study showed that side differences in OB volume correlated to respective differences in odor threshold and odor discrimination, suggesting that OB volume may be dependent on lateralized influences from peripheral input [76]. Altogether, these results suggest that OB volume is regulated, at least partly, by centripetal influences, involving sensory input from the olfactory epithelium. This hypothesis is corroborated by animal studies, which showed that sensory deprivation lead to a decreased OB volume [77,78,79]. Furthermore, in rodents, it has been demonstrated that not only olfactory deprivation leads to a decrease in OB volume but it also leads to decrease in brain-derived neuroptrophic factor (BDNF) expression [80], which has numerous developmental influences on the brain, such as cell differentiation and survival [81].

Nevertheless, results from several studies investigating OB volume of patients suffering from central nervous disease affecting the sense of smell suggest that changes in OB volume might also be due to centrifugal influences. Indeed, patients suffering from temporal lobe epilepsy [82], depression [72], or multiple sclerosis [83] exhibit significantly reduced OB volumes, as compared to healthy controls. Patients suffering from acute major depression showed a significant correlation between OB volume and depression scores [72]. In animal studies, it has been shown that mice exposed to stress have reduced neurogenesis at the level of the SVZ [84]. Hence, it can be speculated that results observed in depressive humans might be due to a reduced neurogenesis, inducing reduced OB volume due to centrifugal influences. 

Interestingly, it has been demonstrated that early blind subjects have superior olfactory abilities and significantly higher OB volume as compared to controls, suggesting that OB plasticity is involved in the compensatory mechanisms between visual deprivation and enhanced olfactory perception [85]. 

In light of these studies, we may reasonably hypothesize that the human OB receives both centrifugal and centripetal influences. This hypothesis might be supported by studies in rodents, which have shown that both centrifugal and centripetal pathways regulate OB activity which itself regulates the recruitment of new neurons. This adult neurogenesis is also directly sensitive to olfactory experience (*i.e.*, sensory deprivation) and to behavioral state (*i.e.*, learning) [23]. However, the specific functional role of centrifugal and centripetal projections in humans as well as the interaction between them and their influence on neurogenesis is not yet known. 

## 5. Conclusions

The OB plays a central role in the processing of olfactory information in humans. Using MRI, several studies have shown that it is a highly plastic structure. Although mechanisms of OB plasticity are well known in animals, the human olfactory system presents several differences as compared to the olfactory system of animals, making it difficult to extrapolate animals results to humans. At present, results of human studies regarding cellular and molecular mechanisms of OB plasticity are controversial. However, based on both microscopic and macroscopic findings, we may reasonably propose that the OB is influenced by both centrifugal and centripetal influences, as well as possible bulbar intrinsic changes. Nevertheless, the exact role and regulation of these different mechanisms remains unclear. Further research is needed to clarify these issues.
